# Supplementary material for: Prediction Model for POstoperative atriaL fibrillAtion in caRdIac Surgery: The POLARIS Score
Source: J Clin Med. 2025 Jan 20;14(2):650. doi: 10.3390/jcm14020650 (PMC11765713; doi:10.3390/jcm14020650)
Supplement: Supplementary file 1 [file jcm-14-00650-s001.zip › jcm-3414249-supplementary.pdf]

Supplemental Material

Figure S1 – Histogram of POAF occurrence by risk score

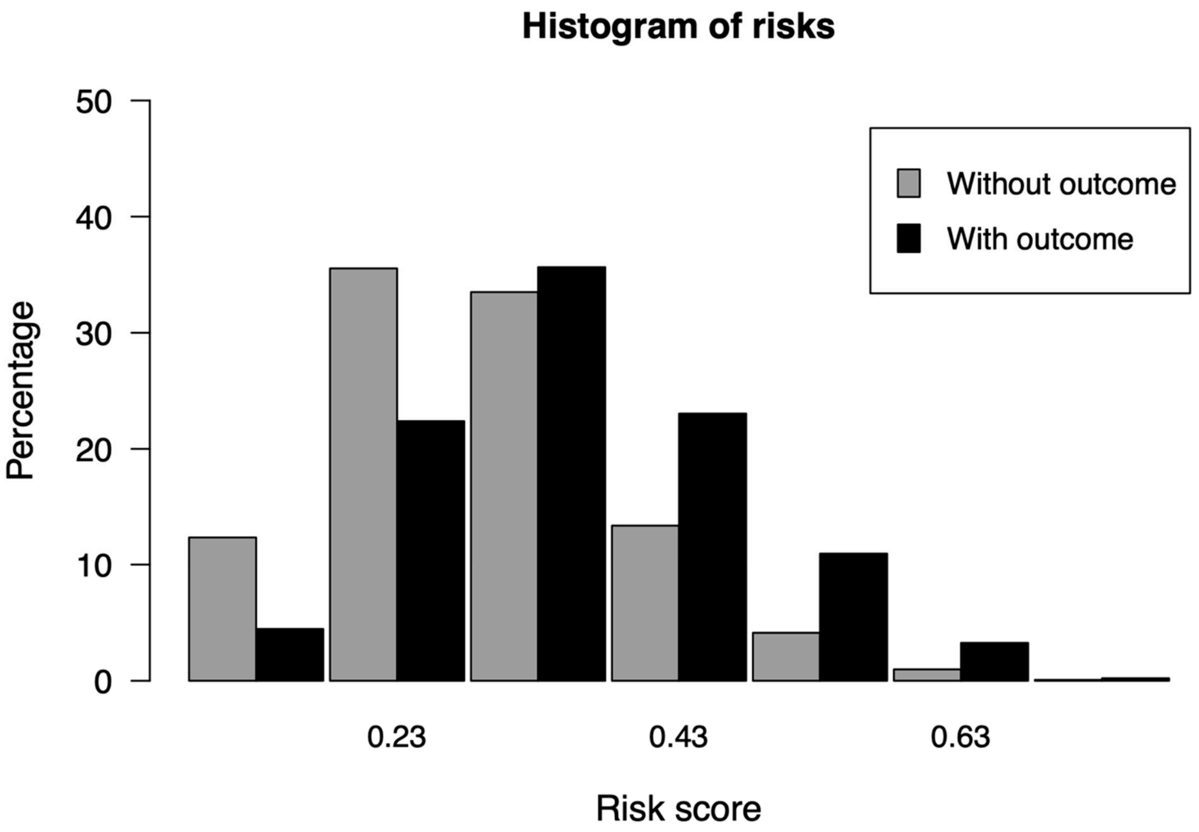

The population distribution based on the presence of POAF (outcome) by risk score is depicted.

Figure S2 – Predictiveness curve of the derivation cohort

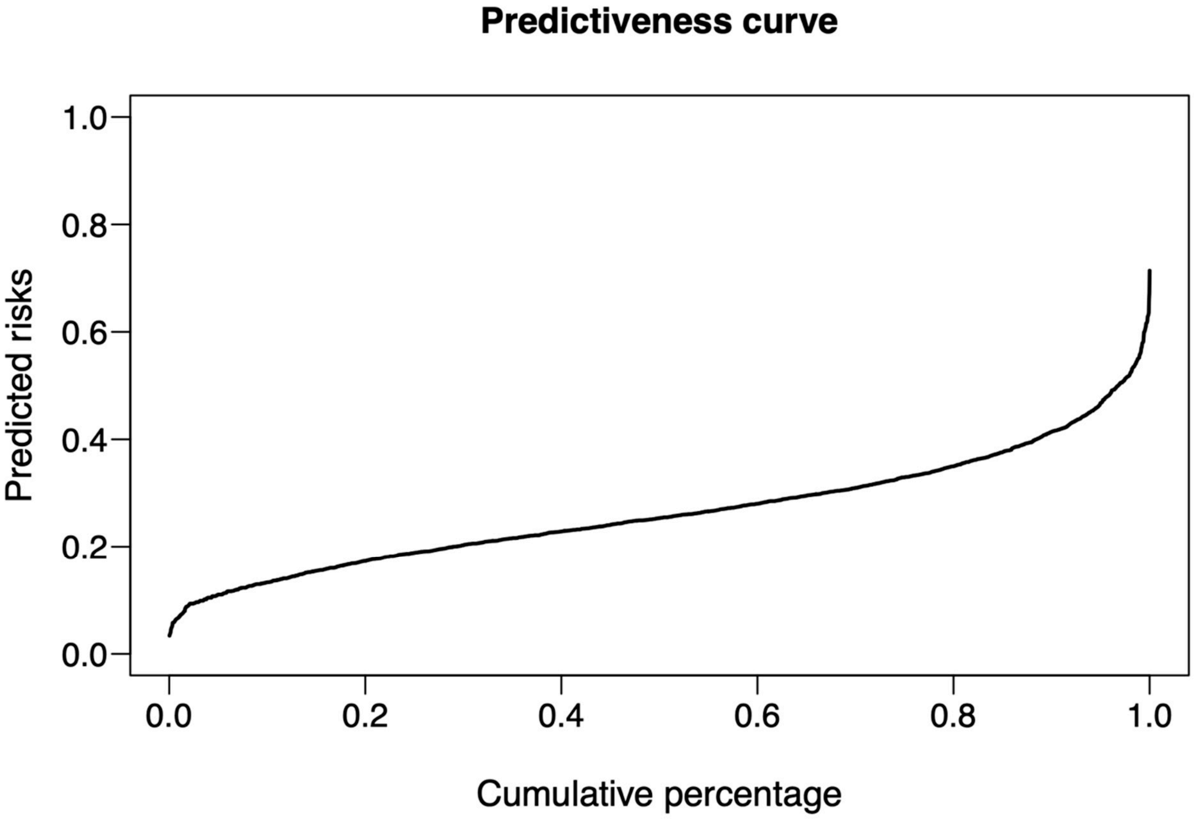

Predictiveness curve of the derivation cohort

Figure S3 – Derivation cohort ROC curve

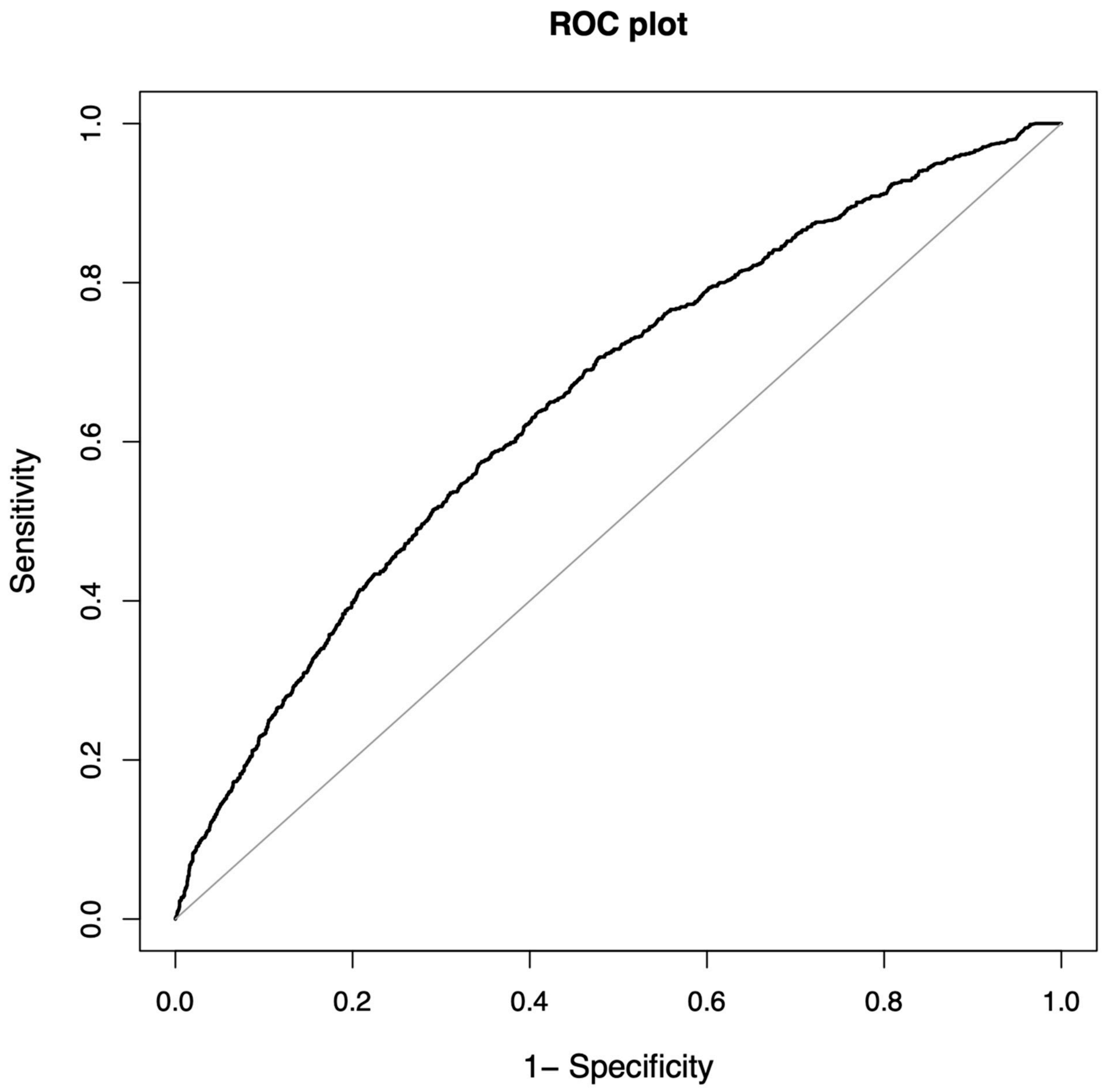

Model discrimination for the derivation cohort was evaluated by using the area under the receiver operating characteristic (ROC) curve.

Figure S4 – Validation cohort ROC curve

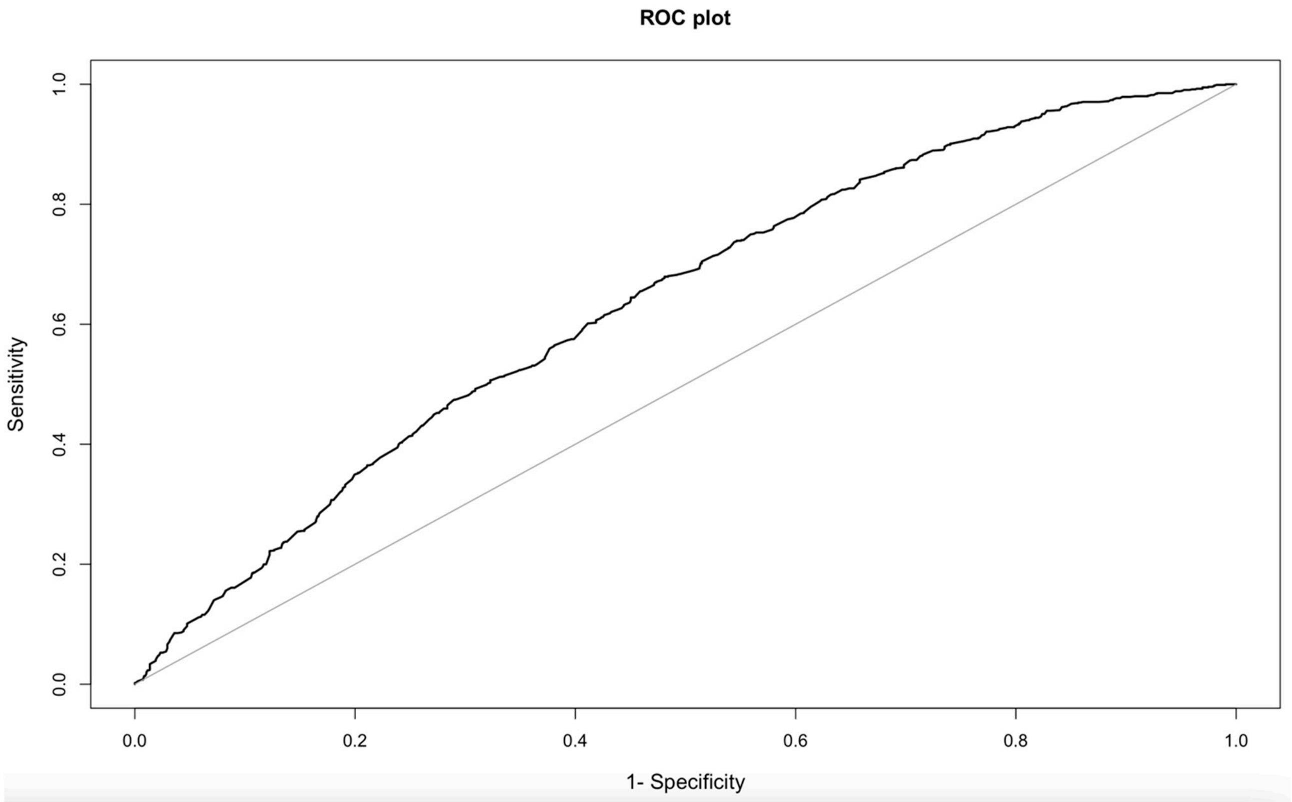

Model discrimination for the validation cohort was evaluated by using the area under the receiver operating characteristic (ROC) curve.

Figure S5 – Predictiveness curve of the validation cohort

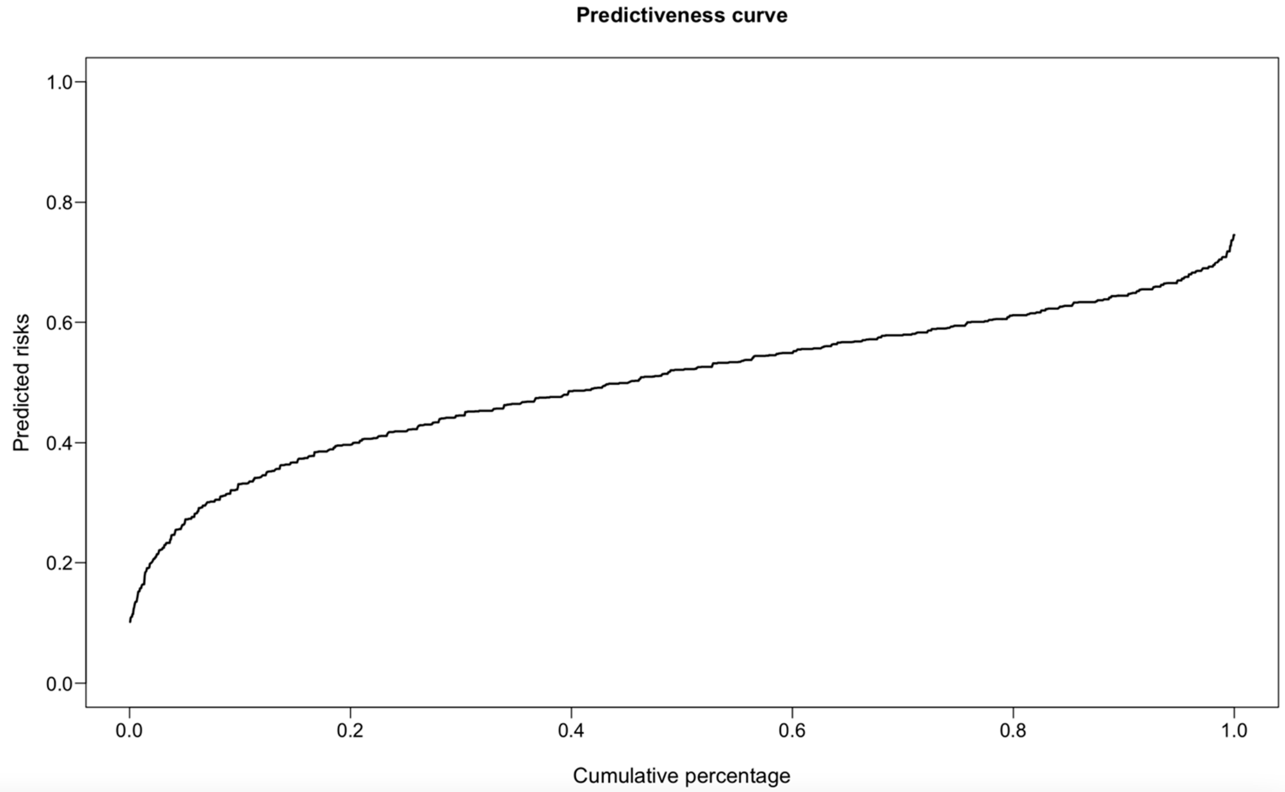

Predictiveness curve of the validation cohort

Table S1 – Creation of the POLARIS score from the ORs

**Variable**	**OR**	**POLARIS score points**
Age >65 years	1.0312	2
Age ≤ 65 years		1
Mitral valve surgery	1.9022	2
Aortic valve surgery	1.4404	1
Minimally invasive	0.6490	0.5
Not minimally invasive		1
PAH	1.3943	1
CKD	1.3065	1
Obesity	1.2036	1

The risk factors were ranked from the “strongest” to the “weakest” and a value ranging from 2 to 0.5 was assigned to each risk factors proportionally in order to obtain the POLARIS score. CKD = chronic kidney disease; OR = odds ratio; PAH = pulmonary arterial hypertension.

Table S2 – Cardioplegia use by POAF outcome

	**No POAF**	**POAF**	**p-value**
Cardioplegia (%)			<0.001
Off-pump	631 (24.8)	150 (16.3)	
Crystalloid	463 (18.2)	245 (26.6)	
Custodiol	429 (16.8)	163 (17.7)	
Warm Hematic	82 (3.2)	13 (1.4)	
Cold Hematic	942 (37.0)	349 (37.9)	

Table S3 – Cardioplegia regression analysis on POAF outcome

	**OR (95% CI)**	**p-value**
Cardioplegia (%)		
Off-pump	REF	
Crystalloid	2.23 (1.76-2.82)	**<0.001**
Custodiol	1.60 (1.24-2.06)	**<0.001**
Warm Hematic	0.67 (0.35-1.19)	0.194
Cold Hematic	1.55 (1.26-1.94)	**<0.001**
